# Treatment of kyphosis in ankylosing spondylitis by osteotomy through the gap of a pathological fracture: a retrospective study

**DOI:** 10.1186/s13018-016-0469-8

**Published:** 2016-11-08

**Authors:** Hongqi Zhang, Zhenhai Zhou, Chaofeng Guo, Yuxiang Wang, Honggui Yu, Longjie Wang

**Affiliations:** Department of Spine Surgery, Xiangya Hospital of Central South University, Xiangya Road 87, Changsha, Hunan 410008 China

**Keywords:** Ankylosing spondylitis, Kyphosis, Pathological fracture, Osteotomy

## Abstract

**Background:**

Surgical interventions are commonly advocated for correcting kyphotic deformities and relieving severe back pain in ankylosing spondylitis (AS) patients. The aim of this study was to evaluate the clinical outcome of osteotomy performed through the gap of a pathological fracture for the treatment of kyphosis in ankylosing spondylitis and to introduce the key points of this novel surgical approach.

**Methods:**

From January 1, 2010, to December 31, 2014, 13 consecutive AS patients who were treated with osteotomy through the fracture gap were retrospectively reviewed. Patients underwent the radiographic assessment of sagittal balance parameters. Visual analog scale (VAS) scores were used to assess improvement in back pain.

**Results:**

The average follow-up time was 2 years and 1 month. The median operation time was 280 min (range, 220–460 min). The mean blood loss was 1100 mL (range, 820–1300 mL). No major acute complications such as death or complete paralysis occurred. There were no neurologic complications or cerebrospinal fluid leaks in any patient. One patient had postoperative wound infection, which subsided after a switch of antibiotics. The global kyphosis Cobb angle of patients decreased from the preoperative 55.8° ± 11.0° to 23.2° ± 6.7° (*P* < 0.001) after surgery. The C7 plumb line was used to assess global balance; its relationship with the posterosuperior corner of the sacrum decreased from 166 ± 37 mm to 111 ± 20 mm (*P* < 0.001). The thoracolumbar kyphosis Cobb angle decreased from 51.0° ± 9.9° to 21.6° ± 11.0° (*P* < 0.001). VAS scores for back pain decreased from 7.2 ± 1.2 to 2.1 ± 1.1 (*P* < 0.001). Lumbar lordosis increased from 5.7° ± 23.2° to 10.5° ± 29.2° (*P* = 0.001).

**Conclusions:**

Osteotomy through the pathological fracture gap is a safe and effective surgical procedure for kyphosis correction and improvement of back pain in AS patients with pathological fractures. A significant kyphosis correction and improvement of back pain can be achieved with this surgical procedure.

## Background

Ankylosing spondylitis (AS) is a chronic inflammatory disease. It always affects the axial skeleton, often starting from the sacroiliac joints and then extending to the upper spine [1]. The interaction between chronic inflammation and the spine is primarily characterized by progressive ossification of the spinal ligaments and facet joints, eventually leading to a fixed and stiff spine [2]. AS is also associated with vertebral osteoporosis [3, 4]. Because of sagittal imbalance of the spine and osteoporosis, pathological fractures can occur, with the mechanism being similar to Chance fracture and seat belt injury [5]. A pathological fracture can occur with minor trauma or even without any trauma, which is different from a general spine fracture, and is most likely in the thoracolumbar junction, a region where tremendous stress is concentrated [6]; the fracture is usually located at the disc level or adjacent to the disc [7].

A pseudoarthrosis usually forms at the fracture site when there is abnormal movement and repeated inflammatory stimuli [8]. Pathological fracture and formation of a pseudoarthrosis progressively increase the kyphotic deformity, with the patient suffering from severe back pain and, in some cases, nerve dysfunction [9]. A progressive kyphosis makes it difficult for the patient to lie down or gaze forward, which can interfere with the performance of daily activities and adversely impact the quality of life. Surgical treatment is the only way to simultaneously relieve back pain and correct kyphosis in AS patients with pathological fractures.

Surgical treatments, including Smith-Petersen osteotomy (SPO or SPOs), pedicle subtraction osteotomy (PSO), vertebral column resection (VCR), polysegmental osteotomy (PO), or any combination of these, are commonly advocated for correcting kyphotic deformities secondary to AS [10, 11]. Presently, three surgical strategies are available for kyphosis correction in AS patients with a pathological fracture or pseudoarthrosis: (1) anterior debridement only; (2) PSO, bone graft fusion, and internal fixation; and (3) a combination of the anterior and posterior approaches [12]. Selection of the operation depends on the extent of ossification of the anterior column and intervertebral disc and the severity of anterior spinal cord compression, apart from many other factors [6]. Despite their advantages, these strategies have concerns due to risky or difficult operation, limited correction angle, and/or increased complications and economic burden.

In our clinical practice, we have developed a new surgical procedure—a posterior osteotomy through the gap of the pathological fracture—that can relieve back pain and correct the kyphotic deformity in patients with AS. In this study, we evaluate the clinical outcomes and correction results in patients undergoing this procedure and discuss the salient features of this novel procedure.

## Methods

### Patients

**Fig. 1:**
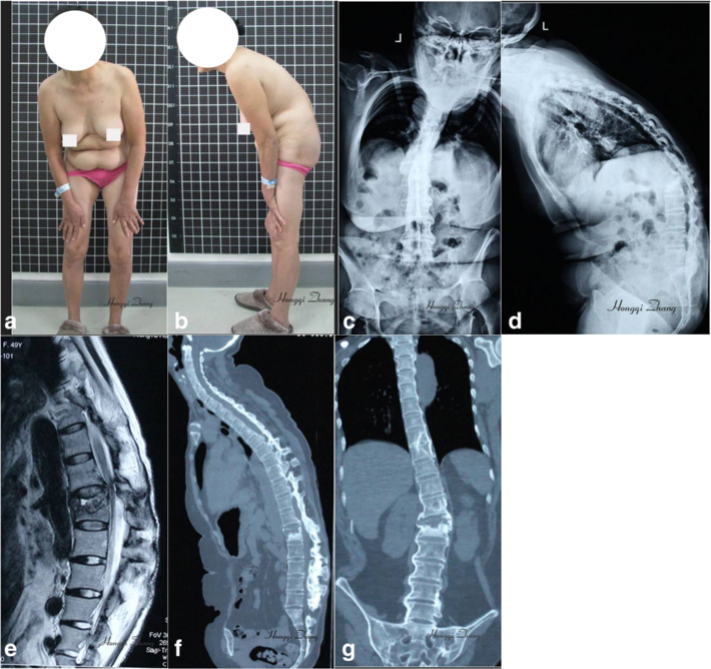
Preoperative imaging findings of a 47-year-old female patient with ankylosing spondylitis. **a**, **b** Photographs show a kyphotic deformity, with the patient having difficulty in holding the head up straight. **c**, **d** Radiographs show the thoracolumbar kyphotic deformity, with a pathological fracture located at L1. **e** MRI image shows destruction of bone and compression of spinal cord. **f**, **g** CT images show the pathological fracture at L1

The study reviewed 13 AS patients with kyphotic deformity and pathological fracture who were treated in our institution, between January 1, 2010, and December 31, 2014. The patients included nine males and four females, with a mean age of 36.8 years (range, 22–52 years). All patients had pathological fractures located at the thoracolumbar junction, including at T9–T11 (*n* = 3), at T11–T12 (*n* = 8), and at T12–L2 (*n* = 2). In this group, one patient had a neurological deficit graded as Frankel D, while the others were either intact or graded as Frankel E. All 13 patients had kyphotic deformities and pathological fractures caused by vertebral osteoporosis and stress concentration (Fig. 1). Patients with low bone mineral density (*n* = 9) received anti-osteoporosis treatment for 1–3 months before and after the operation (Table 1).

**Table 1:** Basic characteristics of patients with ankylosing spondylitis

Mean age (years)	36.8 (22–52)
Male/female	9/4
T9–T11 (*n*)	3
T11–T12 (*n*)	8
T12–L2 (*n*)	2
Low bone mineral density (*n*)	9
Average follow-up time (months)	25 (3–52)

### Surgical techniques

**Fig. 2:**
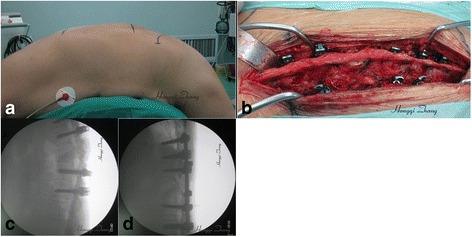
Surgical procedure of osteotomy through the fracture gap in a female patient with ankylosing spondylitis. **a** Photograph shows the position of the patient on the operating table. The patient was flexed in a reverse V shape to accommodate the kyphotic spine and adapt simultaneously to the correction of kyphotic deformity during operation. **b** Photograph shows a hyperplastic osteophyte located at the oseteotomy site. **c**, **d** Intraoperative X-ray fluoroscope was used after inserting the pedicle screws and correcting the kyphotic deformity

The patients were positioned prone on the operating table after general anesthesia. The spine was exposed through a standard posterior midline incision, centering over the predetermined level of the osteotomy. The posterior elements were exposed by subperiosteal dissection as far laterally as the transverse processes. Two or three pairs of transpedicular screws were inserted into adjacent vertebrae, proximal and distal to the osteotomy. Hyperplastic osteophytes were found at the posterior spine at the site of the pathological fracture and the site of osteotomy (Fig. 2).

The spinous process at the osteotomy site was clipped, the hyperplastic osteophytes and vertebral plate were removed, and the fracture gap was probed carefully. After the pathological fracture gap and minor movement were identified, the vertebral plate was resected through the fracture gap and the spinal canal was enlarged. The endorhachis was separated from the spinal canal, and the vertebral plate was then sequentially removed according the preset degree of osteotomy. Titanium rods were truncated and bent according to the physiological curvature and then set and pressed downward. In this process, the middle column became the pivot of the spine, with the anterior column opening and the posterior column closing to correct the kyphosis.

After kyphosis correction, the screws were stressed to shorten the whole three columns and the gap was closed at the pathological fracture in order to facilitate fracture healing. However, a small gap persisted at the osteotomy site, and autogenic iliac bone was used for bone grafting to close the gap. The residual deformity after correction and the closure of the gap at the pathological fracture site were evaluated with fluoroscopy. The beam and the rubber drainage tube were fixed in place, and the incision was closed.

Somatosensory evoked potentials and motor evoked potentials were continuously evaluated during the operation for monitoring spinal cord function. All patients underwent the intraoperative wake-up test after kyphosis correction. Patients were allowed to start walking 2 weeks after surgery and advised to wear a brace for 6 months, until complete bony union had been achieved.

### Radiographic and clinical evaluation

All patients underwent radiographic and clinical evaluation prior to the operation, 2 weeks after the operation, and at follow-up 3 months, 6 months, 1 year, and 2 years after the operation. Radiographic assessment of sagittal balance parameters was performed by standing lateral radiography of the whole spine. Sagittal balance parameters included global kyphosis (GK), lumbar lordosis (LL), thoracolumbar lordosis (TLK), and sagittal vertical axis (SVA; the horizontal distance from a vertical plumb line centered in the middle of the C7 vertebral body to the posterosuperior corner of the S1 endplate). The clinical results were assessed with the visual analog scale (VAS) score.

### Statistical analysis

Statistical analysis was performed using SPSS 17.0 (SPSS Inc., Chicago, IL, USA). All results were reported as means ± standard deviation (SD). The paired sample *t* test was used to compare the preoperative, postoperative, and final follow-up clinical and radiographic data. Statistical significance was set at *P* ≤ 0.05.

## Results

### Operative results

Osteotomy through the fracture gap was successfully performed in all 13 patients. The average follow-up time was 2 years and 1 month. The median operation time was 280 min (range, 220–460 min). The mean blood loss was 1100 mL (range, 820–1300 mL). No major acute complications such as death or complete paralysis occurred. There were no neurologic complications or cerebrospinal fluid leaks in any patient. One patient had wound infection after the operation, which subsided after a switch of antibiotics.

### Radiological results

**Fig. 3:**
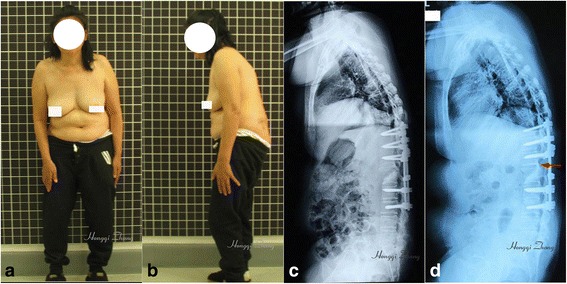
Result of osteotomy through the fracture gap in the female patient with ankylosing spondylitis. **a**, **b** Photographs show satisfactory correction achieved via osteotomy through the pathological fracture gap. **c** Radiograph shows stable internal fixation (without displacement) and corrected kyphosis. **d** Radiograph at follow-up after 1 year shows the closed fracture gap and stable bone fusion achieved at the posterior column

Satisfactory correction of kyphotic deformity was achieved in all patients. In addition, the pathological fracture was healing in all patients at the final follow-up. There were no cases of pseudoarthrosis formation at the osteotomy site or instrumentation failure. No internal fixation loosening, fracture, or correction loss occurred (Figs. 2 and 3).

Radiographic results showed that the postoperative and final follow-up levels of GK decreased significantly compared with the preoperative results (23.2° ± 6.7° and 26.4° ± 9.4° vs. 55.8° ± 11.0°; *P* < 0.001). Similar decreases were found in TLK (21.6° ± 11.0° and 24° ± 8.4° vs. 51.0°; *P* < 0.001). SVA showed smaller decreases (111 ± 20 mm and 87 ± 29 mm vs. 166 ± 37 mm; *P* < 0.001), whereas the opposite changes were observed in LL (10.5° ± 29.0° and 18.8° ± 21.6° vs. 5.7° ± 23.2°; *P* < 0.05; Table 2).

**Table 2:** Radiological assessment of sagittal balance parameters and clinical assessment of preoperation (Pre-OP), postoperation (Post-OP), and at final follow-up (mean ± SD; *n* = 13)

Parameter	Pre-OP	Post-OP	*t* value	*P* value	Final follow-up	*t* value	*P* value
GK (°)	55.8 ± 11.0	23.2 ± 6.7	11.398	<0.001	26.4 ± 9.4	8.733	<0.001
SVA (mm)	166 ± 37	111 ± 20	7.197	<0.001	87 ± 29	8.616	<0.001
TLK (°)	51.0 ± 9.9	21.6 ± 11.0	6.911	<0.001	24 ± 8.4	7.911	<0.001
LL (°)	5.7 ± 23.2	10.5 ± 29.0	−4.674	0.001	18.8 ± 21.6	−2.578	0.024
VAS	7.2 ± 1.2	2.1 ± 1.1	11.813	<0.001	1.9 ± 1.4	12.086	<0.001

### Clinical results

Back pain was obviously improved in all 13 patients. The improvement of VAS scores is shown in Table 2. The postoperative VAS score was markedly lower than the preoperative score (2.1 ± 1.1 vs. 7.2 ± 1.2; *P* < 0.001) (7.2 ± 1.2 vs. 2.1 ± 1.1; *P* < 0.001). Similarly, the final follow-up VAS scores were significantly lower than the preoperative VAS score (1.9 ± 1.4 vs. 7.2 ± 1.2; *P* < 0.001).

## Discussion

### Advantages and disadvantages of various surgical approaches

SPO, PSO, VCR, and PO, or any combination of these, are standardized surgical procedures for correcting kyphotic deformities in AS patients [13, 14]. Because of the increasing vertebral osteoporosis and bony brittleness [15], the ankylosed spine is prone to fracture even after a minor trauma, which is a two- to eightfold increase as compared to non-AS patients [16, 17]. Additionally, the continued movement at the fracture site eventually contributes to the development of pseudoarthrosis [18]. There are three surgical strategies for kyphosis in AS patients with pathological fractures or pseudoarthroses, and the advantages and disadvantages are summarized below:

1. Anterior debridement only: Anterior debridement is especially suitable for eliminating the compression in front of the spine as, for example, in spinal tuberculosis and spinal metastatic carcinoma [19]. The surgeon could have an ideal biomechanical environment via anterior approach [20], and the surgical procedure to eliminate the stress in the front of the spine is much easier [21, 22]. However, a kyphosis correction is difficult to achieve via anterior approach only [23]. Moreover, blood vessels and tissue in front of the spine are easily injured due to ossification of the tissues and ligaments [23, 24].
2. PSO, bone graft fusion, and internal fixation: PSO is modified to treat some fixed sagittal plane deformities in various disease states, including tuberculosis, trauma, and postsurgical conditions [7, 25]. In AS patients, a pseudoarthrosis is liable to be formed at the pathological fracture site. Kyphosis correction and spinal canal decompression can be achieved at the same time by PSO [26]. Nevertheless, limited correction angle is a problem [27]. Additionally, PSO is associated with high risk, and the procedure may not be sufficient to eliminate bone compression in front of the spine [2].
3. Combined anterior and posterior approach: In recent studies, surgeons have demonstrated the value of a combined anterior and posterior approach for kyphosis correction in AS patients with pseudoarthrosis [8, 28, 29]. PSO or SPO (SPOs), bone graft fusion, and internal fixation were adopted in the first stage, followed by anterior debridement in the second stage. This approach could correct the kyphosis and improve symptoms such as back pain and neurologic deficits simultaneously. However, this approach is associated with higher costs, longer hospitalization time, greater operative risks, and more postoperative complications than the one-stage posterior surgical procedure.

Moreover, whether an anterior bone graft is actually needed is still a controversial topic in the field of spinal surgery [10, 30]. Some surgeons deem that the necessity of supplemental anterior fusion for pseudoarthrosis following PSO depends on the extent of the osteotomy closure and the anterior column defect. A pseudoarthrosis is completely cleared after PSO. If the osteotomy site can be completely closed, there is no need to perform an anterior interbody fusion. On the other hand, if the postoperative radiograph demonstrates an anterior column defect with a wide opening at the level of the pseudoarthrosis following PSO, a supplemental anterior fusion must be considered [10, 29]. Qian et al. [29] performed PSO with a supplemental anterior fusion through the pseudoarthrosis in seven AS patients with severe kyphotic deformities. After a mean follow-up of 3 years and 7 months, they reported that the outcome of correction was satisfactory and back pain was obviously improved in all seven patients. However, Chang et al. pointed out that posterior correction and fixation without an anterior support is an effective method for kyphosis correction in AS with pseudoarthrosis. They believed in the superior fusion capacity of AS [31]. In the current study, we have paid more attention to the site of the osteotomy, which is the pathological fracture site. We are also concerned more about the improvement of back pain, fracture healing, and kyphosis correction. SPO, PSO and any kinds of these can be chosen for kyphosis correction according to the predesigned correction angle. Additionally, all the patients underwent one-stage posterior kyphosis correction without an anterior column support, and complete bone fusion and fracture healing were achieved at follow-up. Our data further suggested that a complete bone fusion could be achieved via posterior approach only.

### Key points of the new surgical procedure

In our group of AS patients with kyphotic deformity, the pathological fracture site was chosen as the osteotomy site. Kyphosis correction and improvement of back pain were achieved after the operation, in addition to fracture healing and bone fusion at follow-up. The main features of this operation are as follows:

1. Internal fixation of the spine: Fixed segments provide a stable mechanical environment which is essential for proper correction.
2. Finding the fracture gap: A pseudoarthrosis is always formed in AS patients at the site of a pathological fracture, with compensatory hyperplasia at the fracture site and vertebrae. Therefore, finding the pathological fracture gap is a vital step in this procedure. The main process of finding the fracture gap is to eliminate the hyperplasia of osteophytes in the posterior column and to probe the fracture gap, where a minor movement can be found. Then the fracture gap is enlarged, and dural adhesion is released.
3. Kyphotic deformity correction: After enlarging the fracture gap, the screw–rod system is used to open the anterior spine while closing the posterior spine at the fracture site. This process must be operated slowly and progressively to protect the ossific vessels and tissues in the front of the spine.
4. The screw–rod system for pressing vertebral bodies: In order to decrease the fracture gap, the screw–rod system is used to keep the upper and lower vertebral bodies pressed together, closing the pathological fracture gap. In this process, the middle column becomes the pivot of the spine and the three columns are shortened concurrently. The posterior column is shortened more than the other two columns, thereby avoiding sharp angulation in the sagittal plane and spinal cord shrinkage and preventing excessive opening of the anterior column, which may cause an injury of vessels and tissues in the front of the spine.
5. Autologous iliac crest bone graft: A small gap remains after the kyphosis correction. It is necessary to make the upper and lower vertebral plates coarse for the autologous iliac bone graft. This process contributes to the closure of the fracture gap, which provides support for bone fusion.

### Factors promoting pathological fracture healing

The obvious advantage of this approach is that the fracture can be healed in a short time after the kyphosis correction, without a second-stage anterior bone graft. There are some factors that can promote pathological fracture healing.

1. Fixation: Adjacent segments are fixed by a screw–rod system, and partial stabilization of the spine is increased. Abnormal movement is also decreased at the site of the pseudoarthrosis and fracture. These are necessary conditions for bone healing.
2. Closure of the fracture gap: The fracture gap is decreased with the use of an internal fixation system that stresses the upper and lower vertebral bodies, which is pivotal for bone healing.
3. Change of spine stress line: Before kyphosis correction, shearing force and traction are concentrated at the site of the pathological fracture and the separation traction is mainly concentrated in the posterior column. The spine stress line is improved after a kyphosis correction, and the traction force transforms into stress. In accordance with Wolff’s law, the stress of the fracture site increases and the regional osteogenesis increases [32], which is another factor that promotes bone healing.
4. Autologous iliac bone graft: After kyphosis correction, the autologous iliac bone graft accelerates bone fusion of the residual fracture gap, stabilizes the spine, and provides stable support for the middle and anterior column bone fusion.

### Evaluation of this surgical approach

In the 13 AS patients included in this study, the average kyphosis correction was 31°, which is comparable to that obtained with several other surgical techniques (Table 3) [29, 33, 34]. The correction result was satisfactory, and there was no correction loss during follow-up. At the final follow-up, complete bone fusion had been achieved in all patients. This approach also has its limitations. The degree of kyphosis correction in our study was lower than that which has been achieved with PSO through pseudoarthrosis (Table 3). This procedure is especially applicable in AS patients with a pathological fracture and severe back pain but relatively moderate kyphosis. For AS patients with severe kyphosis, an additional osteotomy, including SPOs or a two-level PSO, is necessary to achieve better correction. A weakness of our study is that it included only a small number of patients. A large randomized controlled study is necessary to accurately evaluate the feasibility, reliability, and complications of this method.

**Table 3:** Results of studies (including ours) that have used osteotomies for correcting kyphosis in ankylosing spondylitis patients with pathological fracture (or pseudoarthrosis)

Author (year)	No. of cases	Surgical method	Single-segment correction (°)	Perioperative complications
Chang (2010) [34]	30	OWO	38	Postoperative pneumonia in 1 patient
				Superficial infection in 1 patient
Kim (2007) [20]	12	SPO + AF or PSO + AF	24 and 31	Intraoperative dural tears in 3 patients
				Leg pain with paresthesia in 2 patients
Qian (2012) [29]	7	PSO through pseudoarthrosis + AF	45	No complications
Our study	13	Osteotomy through pathological fracture gap without AF	31	Superficial infection in 1 patient

*OWO* posterior opening-wedge osteotomy, *SPO* Smith-Petersen osteotomy, *AF* anterior fusion, *PSO* pedicle subtraction osteotomy

## Conclusions

Osteotomy through a pathological fracture gap is a novel and feasible procedure for kyphosis correction in AS patients with pathological fractures. With this method, satisfactory kyphotic deformity correction, successful bone fusion, and obvious improvement of back pain can be achieved simultaneously, without any neurological complications. This surgical procedure is a safe and effective approach for the treatment of AS with pathological fracture and can significantly improve the quality of life.

## Abbreviations

AF: Anterior fusion
AS: Ankylosing spondylitis
GK: Global kyphosis
LL: Lumbar lordosis
OWO: Posterior opening-wedge osteotomy
PO: Polysegmental osteotomy
Post-OP: Postoperation
Pre-OP: Preoperation
PSO: Pedicle subtraction osteotomy
SPO: Smith-Petersen osteotomy
SVA: Sagittal vertical axis
TLK: Thoracolumbar lordosis
VAS: Visual analog scale
VCR: Vertebral column resection
